# Development of appropriateness explicit criteria for cataract extraction by phacoemulsification

**DOI:** 10.1186/1472-6963-6-23

**Published:** 2006-03-02

**Authors:** José Ma Quintana, Antonio Escobar, Inmaculada Aróstegui

**Affiliations:** 1 Unidad de Investigación, Hospital de Galdakao, Galdakao, Vizcaya, Spain; 2 Unidad de Investigación, Hospital de Basurto, Bilbao, Vizcaya, Spain; 3 Departamento de Matemática Aplicada, Estadística e Investigación Operativa, Universidad del País Vasco, Lejona, Vizcaya, Spain

## Abstract

**Background:**

Consensus development techniques were used in the late 1980s to create explicit criteria for the appropriateness of cataract extraction. We developed a new appropriateness of indications tool for cataract following the RAND method. We tested the validity of our panel results.

**Methods:**

Criteria were developed using a modified Delphi panel judgment process. A panel of 12 ophthalmologists was assembled. Ratings were analyzed regarding the level of agreement among panelists. We studied the influence of all variables on the final panel score using linear and logistic regression models. The explicit criteria developed were summarized by classification and regression tree analysis.

**Results:**

Of the 765 indications evaluated by the main panel in the second round, 32.9% were found appropriate, 30.1% uncertain, and 37% inappropriate. Agreement was found in 53% of the indications and disagreement in 0.9%. Seven variables were considered to create the indications and divided into three groups: simple cataract, with diabetic retinopathy, or with other ocular pathologies. The preoperative visual acuity in the cataractous eye and visual function were the variables that best explained the panel scoring. The panel results were synthesized and presented in three decision trees. Misclassification error in the decision trees, as compared with the panel original criteria, was 5.3%.

**Conclusion:**

The parameters tested showed acceptable validity for an evaluation tool. These results support the use of this indication algorithm as a screening tool for assessing the appropriateness of cataract extraction in field studies and for the development of practice guidelines.

## Background

Cataract is very prevalent in developed countries, and the incidence is expected to increase as the result of the aging of the population and increased life expectancies [1]. In addition, cataract extraction is the most common surgical intervention in developed countries [2]. Due to the high prevalence of this condition, clinicians, managers, and health payers must decide who should undergo cataract extraction. Variations in the use of cataract extraction also have been reported, which led to the need to study if overuse or underuse of the procedure was occurring [3-5]. The final goals are to increase the quality of care and the cost-effectiveness in our medical system, which depends on the development of appropriateness criteria to make appropriate decisions.

A way to develop explicit criteria is the methodology developed in the 1980s by the RAND-UCLA group [6]. This is a very popular methodology that has been used in many different diagnostic and therapeutic procedures since then, including cataract extraction. RAND chose cataract extraction as one of the first procedures for which to develop appropriateness criteria [7]. In the1990's, Tobacman et al. applied explicit criteria for cataract extraction following this methodology [8]. Although phacoemulsification has been done since the 1970s and some of the cataract extractions that were reported previously in assessment of appropriateness were performed by phacoemulsification, at that time, phacoemulsification was just beginning to be performed, but now it is the primary surgical technique used for uncomplicated cataract extraction in developed countries [9]. As happens when a new treatment is introduced, the criteria to perform the intervention may change, because new variables should be considered in the decision making process, and some variables that previously were important are no longer so.

The purpose of this study was to update the appropriateness criteria, using the RAND method, for patients undergoing cataract extraction by phacoemulsification exclusively.

## Methods

### Explicit criteria development

The criteria for measuring the appropriateness of cataract surgery were developed according to a previously described explicit method [6], i.e., the RAND appropriateness method, which consists of the following steps.

First, an extensive literature review was performed to summarize existing knowledge on the efficacy, effectiveness, risks, costs, and opinions about the use of phacoemulsification.

Second, from this review, a comprehensive and detailed list of mutually exclusive and clinically specific scenarios (indications) was developed in which cataract surgery by phacoemulsification might be performed. This list contained 765 indications in three categories: simple cataract (cataract with no other ocular pathologies that may affect the visual prognosis), cataract with diabetic retinopathy, and cataract with other ocular pathologies that may affect the visual prognosis. Each indication was specified in sufficient detail that patients within a given indication were reasonably homogeneous. The indications included the following variables. For patients with simple cataract, best-corrected visual acuity in the cataractous eye (three subgroups: ≥0.5, 0.2–0.4, ≤0.1), best-corrected visual acuity in the contralateral eye (three subgroups: ≥0.5, 0.2–0.4; ≤0.1), visual function (four categories: no impairment, glare, difficulty with recreational activities, or difficulty with activities of daily living); surgical complexity of the cataract procedure (three categories: a) No surgical complications or minor complexity anticipated, as the presence of narrow anterior chamber (corneal amplitude-iris <=2), deep-set eyes, extreme myopia without retinal involvement, posterior synechiae, or a small pupil. b) Medium complexity anticipated: Pseudoexfoliation with mydriasis >3 mm and without subluxation of the crystalline lens, dense cataract, poor pupil dilatation (mydriasis >3 mm, according to the dilatation guidelines), vitrectomized eye, poor patient cooperation during examination, and the presence of two or more minor factors. c) High complexity anticipated. Subluxation of the crystalline lens, fibrosis of the anterior capsule of the crystalline lens, brunescent cataract, posterior polar cataract, and the presence of two or more factors of medium complexity); and laterality of cataract (unilateral or bilateral).

For patients with diabetic retinopathy that may affect the visual prognosis and for patients with other ocular pathologies, the same variables were studied plus the anticipated visual acuity after intervention (in three subgroups, ≥0.5, 0.2–0.4; ≤0.1).

The 765 indications resulted from all possible combinations of the variables described and the respective categories. Additional file 1 (Appendix 1) contains a description of the variables and their categories. Cases in which phacoemulsification was performed in combination with other ophthalmic surgical techniques were excluded.

Third, we compiled a national panel of ophthalmologists (doers and non doers of cataract extraction) recognized in the field, the names of whom were provided by their respective medical societies and members of our research team. The panelists were provided with the literature review and the list of indications, and they rated each indication for the appropriateness of performing phacoemulsification, considering the average patient and average physician in the year 2004. Appropriateness was defined as meaning that the "expected health benefit exceeds the expected negative consequences by a sufficiently wide margin to make cataract surgery worth performing."

Ratings were scored on a 9-point scale. Cataract surgery for a specific indication was considered appropriate if the panel's median score was between 7 and 9 without disagreement, inappropriate if the value was between 1 and 3 without disagreement, or uncertain if the median rating was between 4 and 6 or if the members of the panel disagreed. Disagreement was defined as occurring when at least four panelists rated an indication from 1 to 3 and at least another four rated it from 7 to 9. Agreement if less than four panelists rated the indication outside the 3-point region (1–3; 4–6; 7–9) containing the median; and indeterminated if agreement nor disagreement was found. This method did not attempt to force panelists to reach agreement on appropriateness.

The ratings were confidential and took place in two rounds, using a modified Delphi process. The first round was performed by mail before the members of the panel met. The results were collated and presented to the 12 panelists at the 1-day second-round meeting. Each panelist also received the anonymous ratings of the other panelists and a reminder of his or her own ratings. After extensive discussion, the panelists revised the indications according to the above-mentioned definition of appropriateness. Each panelist rated 765 separate indications.

To determine the use of all theoretical indications created in clinical practice, data related to the algorithm variables were gathered for 1,053 patients on a waiting list to undergo cataract extraction by phacoemulsification from six ophthalmologic services at six area hospitals. These data were collected prospectively by the ophthalmologists of each center. The number of theoretical indications used in clinical practice was calculated for each of the three diagnostic groups.

### Statistical analysis

The mean appropriateness ratings of all indications and the mean change from rounds 1 to 2 were calculated for each panelist. The mean difference from each panelist's score for each indication from the panel median of each indication also was measured for both rounds. A "conformity score" [7], describing each panelist's tendency to change his or her ratings in the direction of the round 1 panel median rating also was calculated. This score was defined as a decrease in mean absolute deviation from the round 1 median between rounds 1 and 2. The higher the conformity score, the more the individual's round 2 rating shifted toward the median of the round 1 rating.

We studied the reliability of the 12 panelists scores at 2^nd ^round by performing an intraclass correlation coefficient.

Study of the validity of the explicit criteria: Determinants of appropriateness scores and their contribution to the model explanation were assessed with the least-squares regression model[10], with the median of the panelists' ratings being the dependent variable for each indication, and the variables in the algorithm being the covariates. Ordinal logistic regression also was used, and the classification of the panelists' scores in the categories of appropriate, uncertain, or inappropriate was the dependent variable[11]. Both models were compared regarding the degree of variability explained by each variable. R-square and -2 log L statistics were used, respectively.

Algorithms in decision tree form, which should permit rapid estimation of appropriateness in practice, were compiled from the final results by classification and regression trees (CART) analysis [12]. CART was used to build a classification tree with the appropriateness score of the panel ratings as a dependent variable, as categorical variable (appropriate, uncertain or inappropriate). Misclassification error of the CART, compared to the original panel classification, as the gold standard, was calculated as the ratio of the number of indications erroneously classified by the classification tree divided by the total number of indications.

All statistical analyses were performed using the SAS for Windows, version 8, except for the CART analysis with which we used S-Plus 2000 (MathSoft Inc., 1999) statistical software.

## Results

The same 765 indications were evaluated in the two rounds because no new variables or categories were introduced by the panel of experts in the second round. Agreement among panelists reached 40.1% in the first round and 53.3% in the second round, being then a 0.9% of disagreement at 2^nd ^round. Finally, 32.9% indications were rated as appropriate, 30.1% as uncertain, and 37% as inappropriate (Table 1). Of the three main categories analyzed (simple cataract, cataract with diabetic retinopathy, and cataract with other ocular pathologies), higher agreement was found for simple cataract (57.5%), with a higher appropriateness rate (54.9%). For cataract with diabetic retinopathy, 29.7% indications were rated as appropriate, and 25.2% for cataract with other ocular pathologies (Table 2). Intraclass correlation coefficient among the 12 panelists scores at 2^nd ^round was 0.69.

Table 3 shows the changes in scoring by panelists. Panelists 1 and 6 scored more extremely in round 1 and tended to regress to the mean in round 2, as also can be seen by the higher conformity score. Panelists 7 and 10 did not modify their scores between rounds.

The influence of each variable included in the algorithm was analyzed by linear and logistic regression by category (Table 4). For simple cataract, visual acuity in the operated eye and visual function were the most influential variables. For the other two categories, visual function was the most influential variable followed by the anticipated visual acuity after surgery and the visual acuity in the cataractous eye. Both linear and logistic regression models provided similar results. The type of cataract was not statistically significant in any model, and the contralateral visual acuity was relevant in some cases.

Finally, the criteria developed by the panel of experts were summarized by CART analysis. Figure 1 shows the decision tree for simple cataract. The indications considered appropriate were: 1) visual acuity in the cataractous eye lower than 0.5 and difficulty with activities of daily living, 2) visual acuity of 0.1 or less and glare or difficulty with recreational activities, 3) visual acuity between 0.2 to 0.4 and difficulty with recreational activities (if low or medium surgical complexity), or in cases in which glare is the patient complaint, low surgical complexity, or if medium surgical complexity a contralateral visual acuity between 0.2–0.4, and 4) visual acuity higher than 0.4 if there is difficulty with activities of daily living and low or medium surgical complexity. Globally, cataract extraction was considered inappropriate in patients without impaired visual function with a visual acuity higher than 0.1, or if in the presence of glare or difficulty with recreational activities the visual acuity was higher than 0.4, depending on surgical complexity.

In patients with diabetic retinopathy (Figure 2), the indications considered appropriate were: 1) visual acuity in the cataractous eye of 0.1 or less and difficulty with activities of daily living and an anticipated postoperative visual acuity higher than 0.1, or unilateral cataract with low-to-medium surgical complexity and difficulty with recreational activities or glare and an anticipated postoperative visual acuity between 0.2 to 0.4, or difficulty with recreational activities or glare and an anticipated postoperative visual acuity higher than 0.4. In addition, patients with a visual acuity between 0.2 and 0.4 and an anticipated postoperative visual acuity higher than 0.4 with difficulty with activities of daily living, or with difficulty with recreational activities but with low or medium surgical complexity.

In patients with other ocular pathologies (Figure 3), the indications considered appropriate were: 1) visual acuity in the cataractous eye of 0.1 or lower with glare, with low surgical complexity, and with an anticipated postoperative visual acuity higher than 0.4, 2) difficulty with recreational activities and an anticipated visual acuity higher than 0.1, 3) difficulty with activities of daily living and an anticipated postoperative visual acuity higher than 0.1, 4) visual acuity between 0.2 and 0.4 and an anticipated postoperative visual acuity higher than 0.5 with difficulty with activities of daily living or recreational activities but low surgical complexity.

The classification of the previous decision trees was compared with the original panel scores (Table 5). No indication scored as appropriate by the panel was classified as inappropriate and vice versa. Globally, misclassification error was 0.053. By category, six indications were classified erroneously into the simple cataract group (misclassification error, 0.039), 22 into cataract with diabetic retinopathy (misclassification error, 0.072), and 13 into cataract with other ocular pathologies (misclassification error, 0.042). The numbers of misclassified indications are also indicated in the three figures for each final appropriateness node.

We collected information on 1,053 patients to identify how many of the theoretical indications were used in clinical practice. Globally, 205 of 765 theoretical (26.8%) indications were assigned to 1,053 patients. By category, for simple cataract, 98 of 153 theoretical (64%) indications were assigned to 815 patients with that diagnosis. Of those with diabetic retinopathy, 22 of 306 theoretical (7.2%) indications were assigned to 27 patients. Of those with other ocular pathologies, 85 of 306 theoretical (27.8%) indications were assigned to 211 patients within that category.

## Discussion

RAND appropriateness methodology had been used to create explicit criteria to evaluate the appropriateness of cataract extraction [7,8,13,14]. Nevertheless, as the developers of the RAND methodology pointed out, this methodology cannot capture changes over time with the advent of new diagnostic or therapeutic techniques [15]. This also is the case with cataract extraction. Phacoemulsification is now the most frequently performed technique in developed countries[9] for cataract extraction. Our study incorporates new explicit criteria that can be used with patients undergoing only phacoemulsification.

The work of our panel of experts showed a low disagreement rate among panelists in the second round, compared with other studies in which a similar methodology was used [16]. Also, the intraclass correlation coefficient showed acceptable results. This partially support the reliability of the tool. This can be due to the fact that our panel of experts included only ophthalmologists. The agreement rate was slightly higher for simple cataract than for the other two categories and the proportion of indications scored as appropriate also was slightly higher. This is important because this was the most frequently encountered group in the pilot field study. As shown, previous panels comprised of doctors of one specialty tended to consider more scenarios appropriate than multidisciplinary panels [17]. However, we assembled a mono-specialists panel of expert ophthalmologists because in our health system ophthalmologists alone make the decision to perform cataract surgery. In addition to their interest in our conclusions, our criteria should be available to other physician who may play a role in the referral of these patients to the ophthalmologist so that those patients can be considered for surgery if appropriate.

Our results indicated that among the variables included in the criteria, a few play an important role, as reflected by the linear and logistic regression of all the scores. In addition, the decision trees based on CART analysis used only a few variables. The three important variables were: with simple cataract, the preoperative visual acuity in the cataractous eye, as evaluated by the ophthalmologist; the subjective visual function reported by the patient; and in some cases the surgical complexity. In the other two diagnostic groups (cataract with diabetic retinopathy or with other ocular pathologies), the most important variables were the preoperative visual acuity in the cataractous eye, the subjective visual function and the anticipated postoperative visual acuity in the cataractous eye. Here, again, the preoperative visual acuity in the operated eye was the most important variable, while the visual function was third. These results partially support the face and construct validity of our criteria, since those variables have been identified in different studies as the ones that should play an important role when deciding the appropriateness of cataract extraction[8]. However, studies that did not use the RAND methodology identified the same variables as relevant. Therefore, visual function has been reported by different studies as a key variable from the patient perspective [18]. From the ophthalmologist perspective, the visual acuity in the cataractous eye is the primary variable, followed by others such as the presence of ocular comorbidities or the visual acuity in the contralateral eye [5].

The first study to create explicit criteria following the RAND method developed 1,953 indications during the first round that increased to 2,905 in the second round [8]. The number of diagnoses (ocular comorbidities) included was greater than in our study. In our case, we collected information about the prevalence in clinical practice of all possible ocular pathologies in patients presenting with the need for cataract extraction before we created the indications. We gathered the information from the literature, our administrative databases, and the ophthalmologists of our research team. Since some diagnoses were extremely uncommon, we focused on those most frequently encountered: simple cataract, cataract with diabetic retinopathy, and a new global category called cataract with other ocular pathologies. The last group included some diagnoses that also had been included in the previous RAND studies [8] as separate groups, which led those authors to have a high number of indications. We included them in a single group due to the criteria of our ophthalmologists who considered first, from the standpoint of surgical intervention, that all the diagnostic groups included in this category have in common that affect the anticipated postoperative visual acuity. Second, they considered that there was no other additional criteria-variable- for each of those diagnostic groups who may force us to have them separately.

RAND methodology has some limitations. Among them, the excessive number of indications that were developed[19]. In our case 765 were far fewer than those in previous studies of cataract surgery appropriateness or even in other RAND studies of other non-ophthalmic procedures [16,20]. We considered other diagnostic groups, such as Fuchs' corneal dystrophy or uveitis, but decided not to included Fuchs' corneal dystrophy in this study due to the low prevalence; however, uveitis was included as a surgical complexity. A large number of indications compromised the work of the panel of experts because it forced them to spend considerable time scoring all indications. An additional problem related to the previous one is that the number of theoretical indications used in clinical practice were very few. We found that only 27% of all indications were used, and the percentages varied considerably among the three diagnosis groups, from 73% in simple cataract to 7.2% in cataract with diabetic retinopathy. This means that we asked the panel of experts to score an large number of indications that were unlikely to be present in clinical practice. This can bias the scoring of such indications because no evidence can be found in the literature about the efficacy of phacoemulsification for those indications due to the low prevalence, and because the panelists probably have no experience with them [19]. For this reason, we recommend to future developers of explicit criteria using this methodology to, first, study the prevalence of the different diagnostic groups that lead to the intervention and then develop the theoretical indications that are more likely to be found in clinical practice. Additional criteria also should be the presence of important variability in the use of the procedure or the absence of evidence.

There are different ways to use these explicit criteria. As other authors have done with other procedures, and even with cataract extraction, the explicit criteria can be used for utilization review and health services research studies[14] and can be converted to practice guidelines for use by clinicians, managers, or health payers[21]. However, the criteria should be used cautiously, because as the developers of the RAND method pointed out, other patient circumstances not included in these criteria may play an important role in clinician decision making [22].

In conclusion, this study updates previous work that created explicit criteria for cataract extraction following the RAND methodology. The criteria created are based on variables identified by most studies as relevant, which partially support their validity. Finally, we summarized the results in accurate decision trees, which allow use of the criteria in clinical practice or in the development of practice guidelines.

## Competing interests

The author(s) declare that they have no competing interests.

## Authors' contributions

JMQ conceived of the study, coordinated and participated in the design of the study, and drafted the manuscript. AE participated in the design and helped to draft the manuscript. IA performed the statistical analyses and drafted the manuscript. The IRYSS-Appropriateness Cataract Group participated in the design of the study and helped to draft the manuscript. All authors read and approved the final manuscript.

## Pre-publication history

The pre-publication history for this paper can be accessed here:



## Supplementary Material

Additional File 1 A word document containing the variables included in the appropriateness algorithm. Click here for file

## Figures and Tables

**Table 1:** Agreement and appropriateness judgment of the panel of experts by round.

	**Round 1**				**Round 2**			
	
	**Appropriate**	**Uncertain**	**Inappropriate**	**Total**	**Appropriate**	**Uncertain**	**Inappropriate**	**Total**
**Agreement**	168 (65.)	1 (0.4)	138 (51.1)	307 (40.1)	200 (79.4)	17 (7.4)	191 (67.5)	408 (53.3)
**Indeterminate**	89 (34.6)	219 (92.0)	132 (48.9)	440 (57.5)	52 (20.6)	206 (89.6)	92 (32.5)	350 (45.8)
**Disagreement**	0	18 (7.6)	0	18 (2.4)	0	7 (3.0)	0	7 (0.9)
**Total**	257 (33.6)	238 (31.1)	270 (35.3)	765	252 (32.9)	230 (30.1)	283 (37.0)	765

**Table 2:** Appropriateness and agreement during second round of the panel of experts, by category.

	**Agreement**			
	
**Appropriateness**	**Agreement**	**Indeterminate**	**Disagreement**	**Total**
**Simple Cataract**				
**Appropriate**	69 (82.1)	15 (17.9)	0 (0.0)	84 (54.9)
**Uncertain**	1 (2.3)	39 (90.7)	3 (7.0)	43 (28.1)
**Inappropriate**	18 (69.2)	8 (30.8)	0 (0.0)	26 (17.0)
**Total**	88 (57.5)	62 (40.5)	3 (2.0)	153
**Cataract with Diabetic Retinopathy**				
**Appropriate**	77 (84.6)	14 (15.4)	0 (0.0)	91 (29.7)
**Uncertain**	7 (7.7)	81 (89.0)	3 (3.3)	91 (29.7)
**Inappropriate**	84 (67.7)	40 (32.3)	0 (0.0)	124 (40.5)
**Total**	168 (54.9)	135 (44.1)	3 (1.0)	306
**Cataract with Other Pathologies**				
**Appropriate**	54 (70.1)	23 (29.9)	0 (0.0)	77 (25.2)
**Uncertain**	9 (9.4)	86 (89.6)	1 (1.0)	96 (31.4)
**Inappropriate**	89 (66.9)	44 (33.1)	0 (0.0)	133 (43.5)
**Total**	152 (49.7)	153 (50)	1 (0.3)	306

**Table 3:** Panelists scores during both rounds.

**Panelist**	**Mean Score**			**Mean deviation**		
		
	**Round 1**	**Round 2**	**Mean Change**	**Round 1**	**Round 2**	**Conformity Score**
**1**	3.39	4.31	0.92	1.61	0.90	0.71
**2**	5.44	5.47	0.03	1.23	1.19	0.04
**3**	4.61	4.68	0.07	0.87	0.76	0.11
**4**	4.20	4.38	0.18	0.77	0.68	0.09
**5**	3.93	4.33	0.40	1.08	0.77	0.31
**6**	5.88	5.59	-0.29	1.62	1.22	0.40
**7**	5.16	5.16	0.00	1.68	1.47	0.21
**8**	4.83	4.79	-0.04	1.06	1.04	0.02
**9**	5.97	5.77	-0.20	1.40	1.07	0.33
**10**	5.63	5.61	-0.02	1.85	1.81	0.04
**11**	5.29	5.00	-0.29	0.97	0.62	0.35
**12**	3.97	4.30	0.33	1.03	0.94	0.09

**Table 4:** Contribution of variables to the appropriateness scoring, by chapter.

**Variable by Category**	**Lineal Regression**			**Logistic Regression**		
		
	**R^2^**	**Difference**	**P Value**	**-2 log L**	**Difference**	**P Value**
**Simple Cataract**				210.63		
**Visual Acuity**	0.491	-	<0.001	160.99	49.64	<0.001
**Visual Function**	0.788	0.297	<0.001	117.08	43.91	<0.001
**Surgery Complexity**	0.901	0.113	<0.001	96.41	20.67	0.0003
**Type of Cataract**	0.902	0.001	0.23	94.60	1.81	0.185
**Contralateral Visual Acuity**	0.902	0	0.61	88.36	6.24	0.06
**Cataract with Diabetic Retinopathy**				372.48		
**Visual Function**	0.335	-	<0.001	321.29	51.19	<0.001
**Expected Visual Acuity**	0.487	0.152	<0.001	263.31	57.98	0.006
**Visual Acuity**	0.887	0.4	<0.001	112.94	150.37	<0.001
**Surgery Complexity**	0.913	0.026	<0.001	92.65	20.29	0.005
**Contralateral Visual Acuity**	0.916	0.003	0.006	83.08	9.57	0.02
**Laterality of Cataract**	0.916	0	0.49	83.02	0.06	0.8
**Cataract with Other Pathologies**				345.24		
**Visual Function**	0.397	-	<0.001	260.89	84.35	<0.001
**Anticipated Visual Acuity**	0.554	0.157	<0.001	212.11	48.78	0.03
**Visual Acuity**	0.899	0.345	<0.001	79.60	132.51	<0.001
**Surgery Complexity**	0.913	0.014	<0.001	45.12	34.48	0.004
**Contralateral Visual Acuity**	0.914	0.001	0.1	43.84	1.28	0.55
**Laterality of Cataract**	0.914	0	0.65	41.85	1.99	0.17

R^2^: R Square. Difference: difference in R square after the introduction of the new variable

**Table 5:** Comparison of the classification by the decision trees with the original panel of experts' scores.

	**Panel of Experts**			
	
**Decision trees**	**Appropriate**	**Uncertain**	**Inappropriate**	**Total**
**Simple Cataract**				
**Appropriate**	83	3	0	86
**Uncertain**	1	38	0	39
**Inappropriate**	0	2	26	28
**Total**	84	43	26	153
**Cataract with Diabetic Retinopathy**				
**Appropriate**	87	6	0	93
**Uncertain**	4	75	2	81
**Inappropriate**	0	10	122	132
**Total**	91	91	124	306
**Cataract with Other Pathologies**				
**Appropriate**	73	3	0	76
**Uncertain**	4	91	4	99
**Inappropriate**	0	2	129	131
**Total**	77	96	133	306

Number of indications correctly or incorrectly classified.
