# Supplementary material for: Development of appropriateness explicit criteria for cataract extraction by phacoemulsification
Source: BMC Health Serv Res. 2006 Mar 2;6:23. doi: 10.1186/1472-6963-6-23 (PMC1409777; doi:10.1186/1472-6963-6-23)
Supplement: Additional File 1 — A word document containing the variables included in the appropriateness algorithm. [file 1472-6963-6-23-S1.doc]

**Appendix 1.**

Variables included in the appropriateness algorithm.

1. Diagnostic groups
   1. Simple cataract. Patients who do not present with any other ocular pathology in the operated eye that may affect the visual prognosis. This includes glaucoma controlled by medication or surgery without deteriorating central vision, myopia without retinopathy, and vascular occlusion not affecting central vision.
   2. Cataract with diabetic retinopathy, according to the Early Treatment Diabetic Retinopathy Study classification. This includes nonproliferative diabetic retinopathy, proliferative diabetic retinopathy without high-risk characteristics, cases in which only phacoemulsification will be performed, and the absence of macular edema.
   3. Cataract with other ocular pathologies that may affect visual prognosis. This includes retinopathies: retinal detachments that have been treated surgically, retinitis from any cause; maculopathies: macular degeneration due to toxicity (alcohol), medications, hereditary factors, extreme myopia, age-related macular degeneration; vasculopathies: branch retinal vein occlusion, venous thrombosis; neuropathies: optical neuritis and glaucomatous neuropathy; amblyopia; corneal opacity or corneal dystrophies (except Fuchs’ dystrophy): leukoma without keratoplasty indication.
2. Preoperative visual acuity in the cataractous eye. Best-corrected visual acuity, measured by the ophthalmologist with Snellen optotypes (3 visual acuity categories: ≥0.5; 0.2-0.4; <=0.1).
3. Preoperative visual acuity in the contralateral eye. Similar to the previous category.
4. Visual function. Effect of cataract on patient quality of life in four subgroups:
   1. Unimpaired. Patient reports unimpaired visual function.
   2. Glare (visual discomfort). Visual perception diminished depending on the light intensity (outside or inside).
5. Recreational difficulties. Visual difficulty with activities that do not affect patient autonomy (watching TV, reading, sew, playing).
6. Difficulty with activities of daily living. Activities that affect patient autonomy (as basic ADL such as bathing or eating, or instrumental ADL such as shopping and light household chores), or those that affect their work, which includes glare when driving if this affects the livelihood of the patient.

5. Surgical complexity of the cataract procedure, based on three levels of difficulty:

- 1. No or low complexity anticipated. No surgical complications or the presence of narrow anterior chamber (corneal amplitude-iris <=2), deep-set eyes, extreme myopia without retinal involvement, posterior synechiae, or a small pupil.
  2. Medium complexity anticipated. Pseudoexfoliation with mydriasis >3 mm and without subluxation of the crystalline lens, dense cataract, poor pupil dilatation (mydriasis >3 mm, according to the dilatation guidelines), vitrectomized eye, poor patient cooperation during examination, and the presence of two or more minor factors.
  3. High complexity anticipated. Subluxation of the crystalline lens, fibrosis of the anterior capsule of the crystalline lens, brunescent cataract, posterior polar cataract, and the presence of two or more factors of medium complexity.

1. Anticipated postoperative visual acuity. A subjective preoperative judgment of the ophthalmologist. This is the visual acuity that the patient is expected to achieve postoperatively based on the preoperative ocular examination (3 visual acuity categories: ≥0.5; 0.2-0.4; <=0.1).
2. Cataract (laterality): Unilateral or bilateral cataract.
